# Sentiment Analysis on Tweets about Diabetes: An Aspect-Level Approach

**DOI:** 10.1155/2017/5140631

**Published:** 2017-02-19

**Authors:** María del Pilar Salas-Zárate, José Medina-Moreira, Katty Lagos-Ortiz, Harry Luna-Aveiga, Miguel Ángel Rodríguez-García, Rafael Valencia-García

**Affiliations:** ^1^Departamento de Informática y Sistemas, Universidad de Murcia, 30100 Murcia, Spain; ^2^Universidad de Guayaquil, Cdla. Universitaria Salvador Allende, Guayaquil, Ecuador; ^3^Computational Bioscience Research Center, King Abdullah University of Science and Technology, 4700 KAUST, P.O. Box 2882, Thuwal 23955-6900, Saudi Arabia

## Abstract

In recent years, some methods of sentiment analysis have been developed for the health domain; however, the diabetes domain has not been explored yet. In addition, there is a lack of approaches that analyze the positive or negative orientation of each aspect contained in a document (a review, a piece of news, and a tweet, among others). Based on this understanding, we propose an aspect-level sentiment analysis method based on ontologies in the diabetes domain. The sentiment of the aspects is calculated by considering the words around the aspect which are obtained through* N*-gram methods (*N*-gram after,* N*-gram before, and* N*-gram around). To evaluate the effectiveness of our method, we obtained a corpus from Twitter, which has been manually labelled at aspect level as positive, negative, or neutral. The experimental results show that the best result was obtained through the* N*-gram around method with a precision of 81.93%, a recall of 81.13%, and an *F*-measure of 81.24%.

## 1. Introduction

Diabetes is one of the largest global health emergencies. It is a chronic condition that occurs when the body cannot produce enough insulin or cannot use insulin, and it is diagnosed by observing raised levels of glucose in the blood. The lack, or ineffectiveness, of insulin in a person with diabetes means that glucose remains circulating in the blood. Over time, the resulting high levels of glucose in the blood (known as hyperglycemia) cause damage to many tissues in the body, leading to the development of disabling and life-threatening health complications [1].

Nowadays, it is estimated that there are more than half a million children aged 14 and under living with type 1 diabetes. Also, 415 million adults have diabetes and other 318 million adults are estimated to have impaired glucose tolerance, which puts them at high risk of developing the disease in the future. If this rise is not halted, then the number will exceed 642 million people living with the disease by 2040. According to the International Diabetes Federation (IDF), the top ten countries with the highest number of diabetes are China, India, United States of America, Brazil, Russian Federation, Mexico, Indonesia, Egypt, Japan, and Bangladesh [2].

People suffering from chronic illnesses such as diabetes will have periodic contact with health professionals but they need to have the skills, attitude, and support for self-management of their condition. Therefore, social networks as Twitter are an excellent resource for the patients since they can connect with people who have a similar condition and similar experiences. It provides the environment and the tools for knowledge sharing and peer support. However, the search of opinions is a difficult task; for example, a simple search on Twitter using “diabetes” returns thousands of tweets. It is difficult for users to find relevant information using simple queries. Thus, opinion summarization systems that use sentiment analysis (SA) or opinion mining technologies are needed.

Liu defines SA as “the field of study that analyzes people's opinions, sentiments, evaluations, appraisals, attitudes, and emotions towards entities such as products, services, organizations, individuals, issues, events, topics, and their attributes” [3]. SA can be considered at different levels: at the level of an aspect [4, 5], sentence [6, 7], and document [8]. As mentioned in [9], there is a need for fine-grained approaches to sentiment analysis due to the fact that when a user writes an opinion, it does not mean that the user likes or dislikes everything about the product or service. That is, users give points of view of several aspects that can be positive and negative. This information is important not only for users but also for companies because it supports a decision concerning buying a product, and it can serve as the basis to improve the products and services, respectively [10].

Therefore, we propose a method for aspect-level sentiment analysis that uses ontologies, with the objective of semantically describing relations between concepts in a specific domain. Ontologies allow static knowledge representation and enable knowledge sharing and reuse, thus reducing the effort needed to implement expert systems. Ontologies are currently being applied in several different domains, such as cloud computing [11], natural language interfaces [12], search engines [13], human perception [14], or bioinformatics [15]. One of the reasons for the increasing popularity of this research field is the possibility of providing a shared and common understanding of a domain that can be communicated between people and software applications. In summary, the use of ontologies improves the chance of successfully performing any task related to knowledge and information management.

The remainder of the paper is structured as follows.  Section 2 presents a review of the literature about sentiment analysis in health. The overall design of the proposed approach is described in Section 3.  Section 4 presents the evaluation results concerning the effectiveness of the presented approach. Finally, conclusions and future work are presented in Section 5.

## 2. Related Works

Research in sentiment analysis started in the early 2000s. Since then, several methods to analyze the opinions and emotions from online opinion sources (blogs, forums, or commercial websites) have been introduced. Nowadays a special interest has arisen on the social networks such as Twitter where people share their opinions about several topics [16, 17]. Most of these efforts are based on two main approaches: the semantic orientation (SO) approach and the machine learning approach.

On the one hand, proposals based on SO approach make use of sentiment lexicons such as SentiWordNet [4, 18], iSOL, eSOL [19], and Ml-Senticon [20]. These approaches search each word in the dictionary and assign a positive or negative value. SentiWordNet is the lexicon that has been more widely used in the research community. Despite the fact that promising results have been obtained with lexicons, some proposals have no obtained good results because some words can have different sense (positive or negative) depending on the domain where they are used. To deal with this fact, domain-dependent lexicons have been proposed [21–23]. On the other hand, there are some supervised machine learning based approaches [24–26]. In these works, the classification algorithm needs a training set to learn a model from diverse features of the corpus documents and a testing set to validate the built model from the training set. Among the classification algorithms commonly used in sentiment analysis are Support Vector Machine (SVM) [27–29], Naïve Bayes (NB) [27–30], BayesNet (BN) [31], and Maximum Entropy (MaxEnt) [25, 32]. Furthermore, the performance of the classification algorithms relies on the effectiveness of the feature extraction method used. Thus, several works have focused on feature extraction through the *N*-grams [29] such as unigrams, bigrams, and trigrams. Other proposals evaluate methods based on term frequency-inverse document frequency (TF-IDF), dependency features [33], POS-related features [34], and some cases on their combination [35].

Despite the fact that both approaches, SO and machine learning, have been successfully applied in several domains, they have some disadvantages. On the one hand, a SO approach requires linguistic resources which are scarce for some languages such as Spanish. On the other hand, the supervised machine learning approach requires a large labelled dataset, which is difficult to find in the research community, and the model building process requires a lot of effort and time. In this work, we have followed a semantic orientation approach through SentiWordNet lexicon, which has been successfully applied in several works.

Regarding domains to which the works above are oriented, most of them have validated their approaches in contexts such as movies [4, 19, 24, 25, 31], products [22, 24, 25, 31, 33], and hotels [24]. Furthermore, some of them are oriented to health domain. For instance, Bobicev and Sokolova [30] proposed a method for sentiment analysis on medical forums. They used two classification algorithms, NB and *k*-Nearest Neighbors (KNN). The results show that the NB provides better performance than KNN. Ali et al. [27] proposed a sentiment analysis method on medical forums related to hearing loss. The method is based on supervised machine learning methods such as Naïve Bayes, Support Vector Machine, and logistic regression algorithms. Also, the authors proposed a feature extraction method based on the parts-of-speech information. Aiming to evaluate the performance of the method, a set of experiments were performed using a dataset manually tagged as positive, negative, or neutral. Final results show that the proposed features outperformed the bag of words. In [36], a sentiment analysis framework to detect adverse drug reactions is presented. The authors incorporated features such as *n*-grams, semantic, affective, and emotional. To validate the framework, two datasets collected from a forum post and Twitter were used. Biyani et al. [28] introduced a semisupervised sentiment analysis method that analyzes posts about cancer. The authors used SVM, NB, logistic regression, bagging, and boosting classification algorithms to perform the experiment. Na et al. [37] proposed an aspect-level sentiment analysis method which is applied to drug reviews. They defined a set of rules in order to calculate the polarity value. Also, they developed a review dataset from DrugLib website. In Smith and Lee [29], a supervised sentiment analysis method on clinical reviews is presented. Three classification algorithms SVM, NB, and multinomial NB were used in the experiments. Also, the features were represented as unigrams and bigrams with part-of-speech information. Results show that multinomial NB performed better than SVM. Rodrigues et al. [38] presented SentiHealth (SCH-pt), a tool that allows detecting positive and negative sentiments of cancer patients in Portuguese language. The dataset used for the experiments was collected from Facebook social network. Korkontzelos et al. [39] proposed a method to analyze the sentiment of the Twitter and DailyStrength post about adverse drug reactions. In order to carry out their experiments, a corpus was collected and labelled by expert annotators. Based on the review presented above none of them cover the diabetes domain, which will be addressed in this paper.

Finally, regarding sentiment analysis levels, most works above cited have focused on document-level sentiment classification [28–30, 36, 38, 39], where a polarity positive, negative, or neutral is assigned to the whole document. Also, some approaches have worked in a sentence-level and aspect-level also called feature-level [27, 37]. In this paper, an aspect-level sentiment analysis is addressed because in general few works have been focused at this level, and in the health domain, there is a lack of these approaches. We consider that aspect-level sentiment analysis in health domain is of utmost important since an opinion could contain positive or negative reviews about different aspects of the same drug, medical service, and food, among others.

In summary, our work proposes an aspect-level sentiment analysis approach based on lexicons oriented to the health domain, more specifically to the diabetes domain. In next sections the architecture and its components of the proposed approach are described.

## 3. Approach

**Figure 1:**
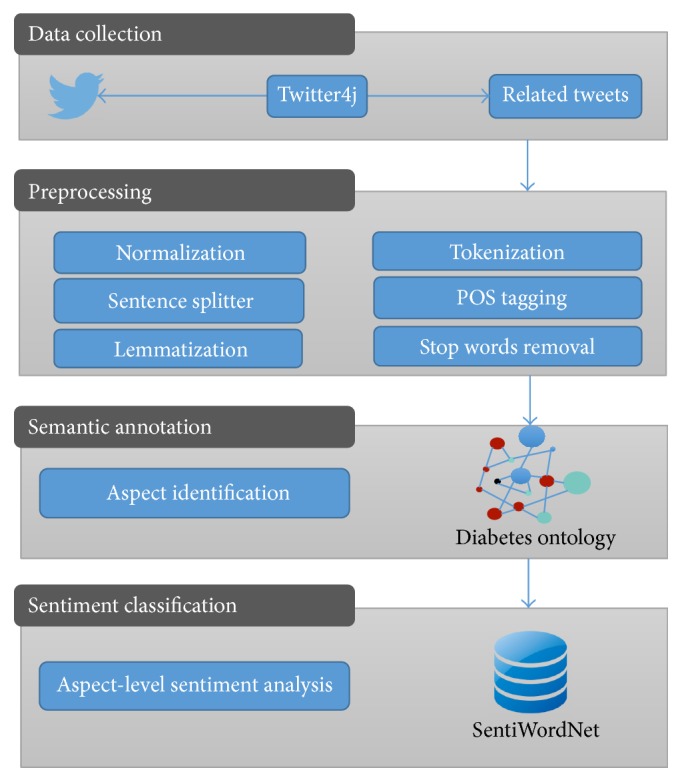
System architecture.

The proposed sentiment classification approach is divided into three main components: (1) preprocessing module, (2) semantic annotation module, and (3) sentiment classification.  Figure 1 shows the architecture of the system. The first module consists in the preprocessing of the corpus to clean and correct the text. The second module involves the detection of aspects by means of the semantic annotation technique. The last module calculates the polarity of each aspect found on the SentiWordNet (SWN) lexicon. A detailed description of the modules contained in the architecture is provided in the following sections.

### 3.1. Preprocessing Module

As can be observed in Figure 1, the preprocessing module involves five processes. The first process, called normalization, consists of three main tasks:

1. The special characters that do not provide important information were removed. For each tweet the following tasks were performed:
  - Remove replies and mentions to other users' tweets, which are represented by strings with @.
  - Remove URLs, that is, strings with “http://”.
  - Remove only the character “#” of Hashtags because the rest represent a word that can be important to the analysis.
2. Correction of spelling errors: in order to perform this task, the Hunspell dictionary [40] has been used.
3. Replace the abbreviations and shorthand notations by their expansions which are not identified by the Hunspell dictionary. Abbreviations are common in tweets due to the fact that there is a limit to 140 characters. Aiming to perform this task, two dictionaries were used: an SMS (Short Message Service) dictionary in order to replace the common abbreviations and shorthand in social networks and a dictionary of the abbreviations about diabetes which was developed by the authors.

The second step, known as tokenization, consists in dividing text into a sequence of tokens, which roughly correspond to “words.” The third step involves assembling the tokenized text into sentences. A sentence ends when a sentence-ending character (., !, or ?) is found which is not grouped with other characters into a token (such as for an abbreviation or number). The fourth step consists in processing a sequence of words and assigning a lexical category to each word. Examples of these categories are NNP (proper noun, singular); VBZ (verb, third person singular present); VBN (verb, past participle); and IN (preposition/subordinating conjunction), among others. The full list of categories is presented in Cunningham et al. [41]. The fifth step refers to the process of mapping words to their base form. For example, the words “buys” and “buying” are mapped onto “buy.” In this work, we used Stanford CoreNLP, a Java annotation pipeline framework that integrates many NLP (natural language processing) tools to carry out the steps two to five.

**Figure 2:**
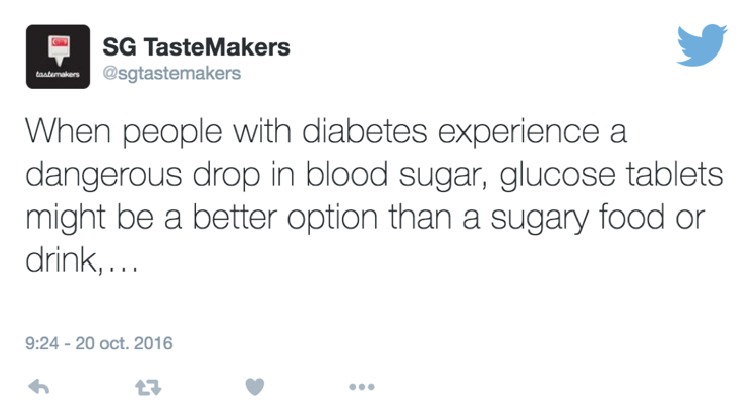
Tweet about diabetes.

Figure 2 shows an example of a tweet about diabetes, while the following part shows the NLP processing result obtained for this example. 

*NLP Techniques Applied to a Tweet*

 
When/WRB/when people/NNS/people with/IN/with diabetes/NN/diabetes experience/NN/experience a/DT/a dangerous/JJ/dangerous drop/NN/drop in/IN/in blood/NN/blood sugar/NN/sugar  ,/,/, glucose/NN/glucose tablets/NNS/tablet might/MD/might be/VB/be a/DT/a better/JJR/better option/NN/option than/IN/than a/DT/a sugary/JJ/sugary food/NN/food or/CC/or drink/NN/drink  ,/,/,…/:/…

As can be seen in the above results, each word in the tweet is tagged with its corresponding lexical category and lemma. For instance, the lexical category of the word “When” is “WRB” which means that it belongs to the “wh”-adverb category. Meanwhile, its lemma does not change with regard to the original word, because it represents the canonical, dictionary, or citation form of the word.

### 3.2. Semantic Annotation Module

This module involves the semantic annotation for each tweet. The semantic annotation is carried out through of a natural language processing (NLP) tool, namely, Stanford NLP, and in accordance with a domain ontology.

Since our objective is to detect aspects concerning the diabetes domain, we have used the diabetes diagnosis ontology (DDO (https://bioportal.bioontology.org/ontologies/DDO)). The main goal of this ontology is to semantically describe relations between concepts in the diabetes domain.

**Figure 3:**
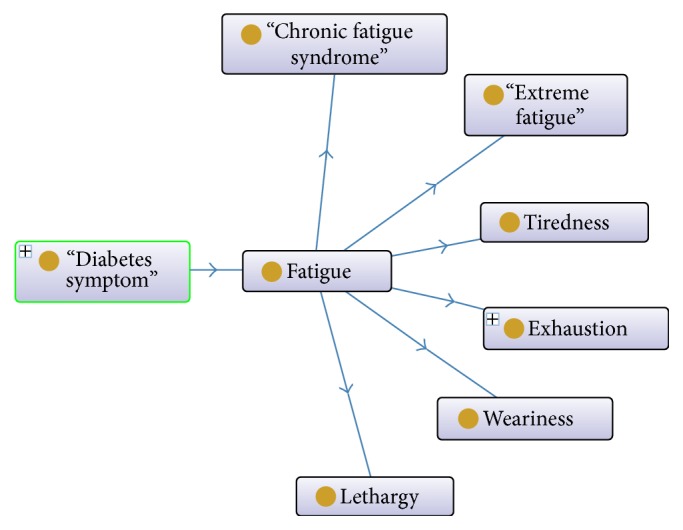
Excerpt from DDO ontology.

DDO ontology extends of ontology for general medical sciences (OGMS (https://www.bioportal.bioontology.org/ontologies/OGMS)). OGMS provides a set of classes related to patients, diseases, and diagnoses. Also, it follows the paradigm of basic formal ontology (BFO (http://www.ifomis.uni-saarland.de/bfo/)). DDO represent a detail every facet of the diabetes disease. Among the main aspects included are clinical presentation, physical manifestation, diagnosis, treatment, laboratory tests, and course of development. This ontology is described using the Web Ontology Language (OWL) 2. The ontology defines 6444 classes, 6821 subclass axioms, 6 data type properties, and 42 object properties [42]. An extract of the ontology is shown in Figure 3.

The five outstanding concepts of the ontology proposed are described as follows:

- Diabetic complication: this represents complications related to diabetes such as chronic disease, food disease, and cardiovascular disease, to mention but a few.
- Drug: this represents medicines that affect blood glucose levels such as analgesics, antiasthmatic, autonomic, and antihistamine.
- Laboratory test: this represents the laboratory tests to diagnose diabetes such as insulin antibodies, HbA1c, serum ketone, and total cholesterol.
- Physical examination: this subsumes the physical signs that help to take a decision of the diagnostic, for instance, smoking, blood pressure, physical activity, and alcohol drinking, to mention a few.
- Diabetes symptom: this subsumes the manifestations related to diabetes such as diarrhea, fatigue, edema, nausea, and foot symptom.

### 3.3. Sentiment Classification

In this module, the sentiments of the aspects of each tweet are obtained. We relied on a proximity approach, where the words that are close to each aspect are obtained. This process has been carried out using the “*N*-gram after,” “*N*-gram before,” and “*N*-gram around” methods, which have already been studied in the literature [4, 10].

- *N*-gram before method: it consists in the extraction the* N*-gram words before the aspect in the tweet.
- *N*-gram after method: it consists in the extraction the* N*-gram words after the aspect in the tweet.
- *N*-gram around method: it consists in the extraction the* N*-gram words before the aspect and the* N*-gram words after the aspect in the tweet.

*N*-gram refers to the number of words near of the aspect that are considered for the sentiment identification. In this work, we consider* N*-gram from 2 to 6.

The polarity of the closest words to the aspect identified is calculated by using SentiWordNet (SWN) [43]. SWN is a lexical resource that associates three numeric values with each synset of WordNet: positivity, objectivity, and negative. The sum of all three values is equal to 1. Each entry in SWN has multiple senses; for example, the word “better” has sixteen senses in SWN (four that belong to the category “adjective,” seven that belong to the category “noun,” two that belong to the category “adverb,” and three that belong to the category “verb”). In order to address this issue, we have used Babelfy. Babelfy is a multilingual tool for Word Sense Disambiguation (WSD) [44].

After disambiguating the sense using Babelfy, we retrieve the SentiWordNet scores for the matching sense of that word. In accordance with the example presented in Figure 2, the word “better” is identified as* “(comparative of “good”) superior to another (of the same class or set or kind) in excellence or quality or desirability or suitability; more highly skilled than another”* in Babelfy, which corresponded in SWN to the adjective ID “00230335”, with the following positive, negative, and neutral scores: ScorePosSwn = 0.875, ScoreNegSwn = 0, and ScoreNeuSwn = 0.125.

The positive, negative, and neutral scores of each aspect are then calculated using the following equations:

(1) ScorePosai=∑w∈waiScorePosSwnw,

(2) ScoreNegai=∑w∈waiScoreNegSwnw,

(3) ScoreNeuai=∑w∈waiScoreNeuSwnw,

where* ScorePosSwn*,* ScoreNegSwn*, and* ScoreNeuSwn* are the positive, negative, or neutral scores obtained by means of SWN for each word (*w*), which belongs to the set of words (*wf*_*i*_) obtained using the three* N*-gram methods, for each aspect (*a*_*i*_).

An aspect is therefore positive if ScorePos(*a*_*i*_) > ScoreNeg(*a*_*i*_) and ScorePos(*a*_*i*_) > ScoreNeu(*a*_*i*_). In contrast, it is negative if ScoreNeg(*a*_*i*_) > ScorePos(*a*_*i*_) and ScoreNeg(*a*_*i*_) > ScoreNeu(*a*_*i*_). Finally, it is defined as neutral if ScoreNeu(*a*_*i*_) > ScorePos(*a*_*i*_) and ScoreNeu(*a*_*i*_) > ScoreNeg(*a*_*i*_).

## 4. Experiments 

In this work, we carried out a set of experiments to measure the effectiveness of the approach about aspect-level sentiment analysis. A detailed description of these experiments is provided below.

### 4.1. Data

The set of experiments performed in this work involved the use of one dataset concerning diabetes. The corpus consists of a total of 900 tweets, which were collected use Twitter4J, a Java library that facilitates the usage of Twitter API. The tweets were manually tagged at aspect-level to obtain the baseline results to evaluate our proposed method. It should be mentioned that this task was performed in a period of 4 months by a group of three experts. The process involves reading the tweets and identifying all aspects and associated polarities (positive, negative, or neutral).

### 4.2. Evaluation and Results

In order to measure the performance of our method, we have used well-known metrics: precision, recall, and *F*-measure. Precision (4) represents the proportion of predicted positive cases that are real positives. On the other hand, Recall (5) is the proportion of actual positive cases that were correctly predicted as such. *F*-measure (6) is the harmonic mean of precision and recall [10]:

(4) Precision=True  PositivesTrue  Positives+False  Positives,

(5) Recall=True  PositivesTrue  Positives+False  Negatives,

(6) F-measure=2∗Precision∗RecallPrecision+Recall.

The first experiment consists in measuring the performance of our system to identify aspects correctly (see Table 1). The process involved comparing the results obtained by the system with the labelled aspects as baseline to obtain the number of aspects correctly identified. As can be seen in Table 1, our system obtained encouraging results for aspect identification with a precision of 85.71%, a recall of 80.00%, and an *F*-measure of 82.75%.

These results indicate that the use of a domain ontology in the process for aspect identification allows getting good results. However, the results also show that several aspects identified by the expert user were not identified by the system, which we attribute to the lack of some concepts and synonyms in the ontology.

The second experiment involves evaluating the system performance for aspect-level sentiment analysis. As mentioned previously, three* N*-gram methods (*N*-gram before,* N*-gram after, and* N*-gram around) were used to identify the correct polarity of an aspect. For each* N*-gram method, values from 2 to 6 were considered aiming to discover the best result. The polarity obtained for each aspect by the system was compared with the manual results.  Table 2 shows the aspect-level sentiment analysis results obtained by means of the three* N*-gram methods.

As regards the “*N*-gram before method,” the best result is obtained with* N*-gram = 3 with a precision of 69.74%, a recall of 69.33%, and an *F*-measure of 69.42%. Quite the reverse, the* N*-gram = 2 obtained the worst results with a precision of 63.51%, a recall of 62.00%, and an *F*-measure of 62.22%.

In the case of the “*N*-gram after” method, the results are better than those obtained using the “*N*-gram before” method. The best result (a precision of 78.62%, a recall of 78.20%, and an *F*-measure of 78.31%) is obtained with an* N*-gram = 3, as occurred with the “*N*-gram before” method. Conversely, the worst result is also obtained with* N*-gram = 2 (a precision of 73.93%, a recall of 73.33%, and an *F*-measure of 73.48%).

Regarding the “*N*-gram around” method, it provides higher results than the obtained ones by the other two methods (*N*-gram before and* N*-gram after), signifying that it is important to consider both the previous and next words of the aspect identified. The best result is also obtained with* N*-gram = 3 with a precision of 81.93%, a recall of 81.13%, and an *F*-measure of 81.24%. This means that the best results are obtained when considering the three words that precede and follow each aspect contained in the tweet.

### 4.3. Discussion of General Results

The results show that the proposed approach provides encouraging results for aspect identification and aspect-level sentiment analysis of tweets about diabetes in the English language.

**Figure 4:**
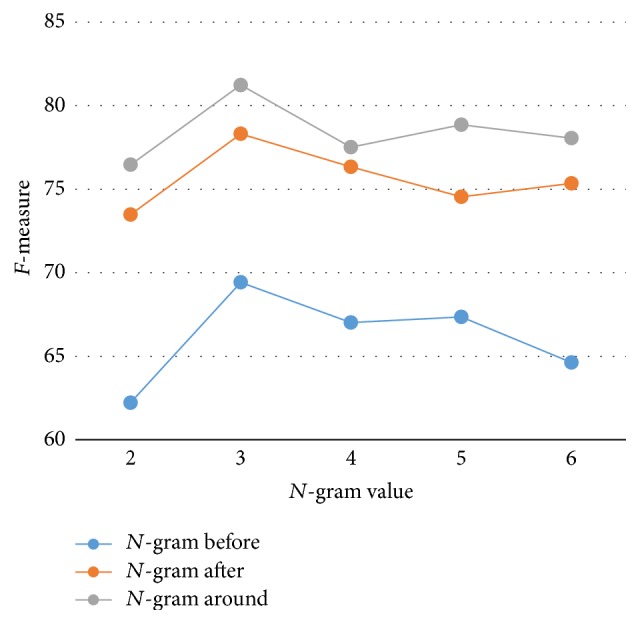
Aspect-level sentiment analysis with* N*-gram methods.

Regarding the* N*-gram methods (see Figure 4), the “*N*-gram around” achieves the best results with a precision of 81.93%, a recall of 81.13%, and an *F*-measure of 81.24%. Thus, the “*N*-gram around” method represents an optimal means to carry out the sentiment analysis of tweets concerning the diabetes domain in the English language.

As regards the* N*-gram values, the* N*-gram after,* N*-gram before, and* N*-gram around methods obtained the best results with an* N*-gram = 3. This means that, for* N*-gram before, the best result is obtained when the three previous words are used to identify the sentiment of the aspect. Quite the reverse, for* N*-gram after, the best result is obtained when the next three words are used to identify the sentiment of the aspect. Thus, in the case of* N*-gram around, the best result is obtained when considering the three words that precede and follow each aspect contained in the tweet.

On the other hand, despite the fact that the results obtained for aspect identification are encouraging, we consider that our proposal can be improved. An issue that we detected was that the system does not identify some synonyms. For example, some tweets contain the concept “oral hypoglycemic,” which is contained in the ontology; however, some other tweets contained the synonym “oral antihyperglycemic,” which is not detected for the system. These results are highly dependent on the ontology. Therefore, we are considering an ontology evolution strategy that allows the ontology used to grow in size through the addition of new concept or some new properties such as synonyms.

Another issue detected was that some shorthand and abbreviations about diabetes were not replaced by their expansions. For instance, in Twitter “Hypo” is used instead of “Hypoglycemia” and “carbs” instead of “carbohydrates”; therefore, we plan to enrich the dictionary with diabetes jargon, abbreviations and terminology frequently used in social networks.

### 4.4. Comparison with Related Work

Table 3 shows a comparison of some related works in the health domain in terms of precision (*P*), recall (*R*), *F*-measure (*F*1), and accuracy (ACC). As can be seen, the main topics addressed are adverse drug reactions [36, 39] and cancer posts [28, 38]. Also, only a few are focused on clinical opinions [29] and hearing loss [27]. Furthermore, the English language is the most used [27–30, 36, 37, 39], although the work [38] presented a proposal based on Portuguese. Regarding the sentiment analysis level, the document-level is the most attended [28–30, 36, 38, 39]. Conversely, sentence-level and aspect-level have been little studied [27, 37].

Table 3 shows that our proposal has obtained encouraging results outperforming several proposals with a precision of 81.93, recall of 81.13, and *F*-measure of 81.24. However, to carry out a fair comparison between proposals is a difficult task for two reasons: (1) The approaches are oriented to different sentiment analysis levels, mainly at document-level, and our proposal is based at aspect-level; (2) the proposals are based on different approaches (machine learning or semantic orientation), topics, and languages, so that the linguistic resources used differ in size, context, and language, which makes the comparison between the proposals difficult. In this sense, to perform a proper comparison the use of the same corpus in all evaluations is needed.

## 5. Conclusions

This paper presented an aspect-level sentiment analysis approach in the diabetes domain for the English language. Our approach uses an ontology to effectively detect aspects concerning diabetes in tweets. We have also presented a set of experiments aiming to validate our system for aspect identification and aspect-level sentiment classification. The results show that the “*N*-gram around” method obtained the best results with a precision of 81.93%, a recall of 81.13%, and an *F*-measure of 81.24%.

Despite its contributions, this study has certain limitations. First, the proposed approach is only able to deal with tweets in English language. This is a disadvantage due to the fact that vast amount of information about diabetes is available in other languages. Therefore, we plan to apply this method to other languages such as Spanish. Second, the experiments showed that the general sentiment lexicon is not enough suited for capturing the meanings in health texts. We are therefore interested in the construction of a domain-specific sentiment lexicon such as that presented in [22, 23]. Third, our approach requires an ontology that models the domain to identify the aspects. However, it is difficult to find established ontologies and their manual development is highly time and effort consuming task; therefore, we plan to adopt approaches such as [45, 46] to the automatic or semiautomatic creation of ontologies, in order to obtain the domain concepts and relations from a set of documents related to a specific domain.

## Figures and Tables

**Table 1:** Aspect identification.

Precision	Recall	*F*-measure
85.71	80.00	82.75

**Table 2:** Aspect-level sentiment analysis results obtained with the three *N*-gram methods.

*N*-gram value		*N*-gram before	*N*-gram after	*N*-gram around
2	*P*	63.51	73.93	77.20
	*R*	62.00	73.33	76.33
	*F*1	62.22	73.48	76.46
3	*P*	69.74	78.62	**81.93**
	*R*	69.33	78.20	**81.13**
	*F*1	69.42	78.31	**81.24**
4	*P*	67.57	76.71	78.25
	*R*	66.93	76.20	77.40
	*F*1	67.02	76.34	77.52
5	*P*	67.91	74.95	79.60
	*R*	67.27	74.40	78.73
	*F*1	67.35	74.54	78.86
6	*P*	65.40	75.77	78.81
	*R*	64.53	75.20	77.93
	*F*1	64.62	75.34	78.06

**Table 3:** Comparison with related work.

Proposal	Level	Language	Domain	*P*	*R*	*F*1	ACC
Bobicev & Sokolova [30]	Document	English	Health reviews	52.70	54.10	51.80	79.90
Ali et al. [27]	Sentence	English	Hearing loss	68.80	68.60	68.50	—
Sharif et al. [36]	Document	English	Adverse drug reactions	—	—	—	78.20
							79.30
Biyani et al. [28]	Document	English	Cancer post	84.40	84.30	84.40	—
Na et al. [37]	Aspect	English	Drug reviews	79.00	78.00	78.00	78.00
Smith and Lee [29]	Document	English	Clinical reviews	81.58	83.53	83.52	83.53
Rodrigues et al. [38]	Document	Portuguese	Cancer post	59.36	61.52	59.08	71.00
Korkontzelos et al. [39]	Document	English	Adverse drug reactions	87.19	78.93	82.86	—
				78.51	68.59	72.94	
Our proposal	Aspect	English	Diabetes	81.93	81.13	81.24	—
